# A phase I, open-label study of intravenous human dental pulp stem cells (NestaCell®) at two dose levels in patients with Huntington’s disease

**DOI:** 10.1186/s13287-025-04703-w

**Published:** 2025-11-04

**Authors:** Joyce Macedo Sanches Fernandes, Eduardo Pagani, Cristiane Valverde Wenceslau, Leandro Hideki Ynoue, Luciana Ferrara, Irina Kerkis

**Affiliations:** 1 Programa de Pós-Graduação Hospital das Clínicas, Faculdade de Medicina, Universidade Estadual de Campinas (UNICAMP), Campinas, SP 13083-872 Brazil; 2 Azidus Brasil, Valinhos, SP 13271-130 Brazil; 3 Cellavita Pesquisas Científicas Ltda., Valinhos, SP Brazil; 4 Laboratório de Genética, Centro de Desenvolvimento e Inovação (CDI), Instituto Butantan, São Paulo, SP 05503–900 Brazil

**Keywords:** Huntington’s disease, Stem cells, Unified Huntington’s disease rating scale, Dental pulp stem cell

## Abstract

**Background:**

Huntington’s disease (HD) is a progressive neurodegenerative disorder with no approved disease-modifying therapies. Human dental pulp stem cells (hDPSCs) offer potential therapeutic benefits due to their neurogenic, neurotrophic, and immunomodulatory properties. This prospective, open-label, single-centre, first-in-human clinical trial evaluated the safety, tolerability, and preliminary efficacy of intravenous hDPSC in patients with HD.

**Methods:**

Six male patients with HD received intravenous infusions of hDPSCs in two dosage cohorts: three patients received 1 million cells/kg, and three received 2 million cells/kg. The treatment protocol consisted of cycles of three infusions at monthly intervals followed by subsequent administration cycles every six months, as per a protocol amendment based on the initial favourable safety outcomes. The total number of infusions ranged from 4 to 26 over the five years. During the first year, all patients underwent intensive multiparametric monitoring in an intensive care unit (ICU) for 48 h after each infusion.

**Results:**

No adverse events occurred during the 48-h ICU monitoring or within 15 days post-infusion. Of 41 treatment-emergent adverse events (TEAEs) reported during follow-up, 35 were judged unrelated to the hDPSCs, mainly reflecting disease progression or incidental findings. Six treatment-emergent adverse events (TEAEs) were considered treatment-related, involving transient changes in hair pigmentation or regrowth. One patient discontinued due to a serious adverse event—lung cancer arising from a pre-existing pulmonary nodule identified at enrolment. Genetic analysis of the excised tumour showed no evidence of investigational product engraftment, supporting its non-tumorigenic nature. The same patient experienced a severe depressive episode lasting approximately 93 days; the relationship to treatment was considered uncertain. Minor, clinically insignificant fluctuations in CD4/CD8 lymphocyte counts and cytokine levels were observed. Preliminary efficacy analyses indicated potential stabilisation of disease progression, particularly in the Unified Huntington’s Disease Rating Scale (UHDRS), Total Motor Score (TMS) and Total Functional Capacity (TFC).

**Conclusions:**

hDPSCs infusions were well tolerated and exhibited a favourable safety profile, even with prolonged exposure and follow-up. These findings support the continued clinical development and warrant further investigation in more extensive trials to assess therapeutic efficacy in Huntington’s disease.

**Trial registration:**

This study was registered on April 4, 2016, at ClinicalTrials.gov (identifier: NCT02728115; https://clinicaltrials.gov/study/NCT02728115).

## Background

Human dental pulp stem cells (hDPSCs) represent a promising resource for cell-based therapies due to their multipotency, immunomodulatory capabilities, and ease of isolation [1–4]. Comparative analyses between hDPSCs and other mesenchymal stem cell (MSC) sources, such as those derived from umbilical cord and menstrual blood, suggest that hDPSCs exhibit distinct advantages in terms of proliferation rates, differentiation potential, and accessibility [3, 5].

Numerous studies have demonstrated the potential of hDPSCs in regenerative medicine, particularly in neurological applications, due to their neurogenic and neurotrophic properties [4, 6–11]. hDPSCs have demonstrated efficacy in animal models for spinal cord injury [12, 13], stroke-related chronic disabilities [9], Parkinson’s disease [14, 15], retinal degeneration [6, 16], osteoarthritis [17, 18], wound healing and muscle regeneration [19], bone regeneration and repair [20], and neurodegenerative disorders [4, 8, 21]. These beneficial effects are primarily attributed to the secretion of neural growth factors and cytokines that modulate inflammation, promote tissue regeneration, and support neuronal survival and repair [22–25]. Additionally, their regenerative and immunomodulatory properties have sparked interest in their potential therapeutic use in treating COVID-19 [25].

The clinical translation of hDPSC-based therapies has been progressively explored, with a focus on safety, feasibility, and therapeutic efficacy [2, 9, 21, 26, 27]. Recent clinical trials have highlighted their potential in treating neurological conditions, including chronic stroke [9], retinal degenerative disorders [16], and other degenerative diseases [2, 21]. Nevertheless, standardisation in the isolation, characterisation, and clinical application of hDPSCs remains critical for successful translation from research to therapy [2, 26–28]. Challenges such as cellular heterogeneity, donor variability, and optimisation of culturing conditions remain essential for achieving reliable therapeutic outcomes [26, 27]. NestaCell® is a novel product consisting of cryopreserved hDPSC suspended in sterile saline, expressing nestin (a protein that supports axon growth) and secreting high levels of brain-derived neurotrophic factor (BDNF). After thawing, these cells are administered intravenously and can migrate to brain tissues. Due to the low expression of HLA-DR antigens, NestaCell® is safe for heterologous use without the need for immune suppression. Transcriptome analysis of different batches revealed that NestaCell® expresses 375 unique genes, promoting axon growth and guidance, distinguishing it from other MSCs [29, 30].

Preclinical studies involving the intravenous administration of NestaCell® demonstrated promising outcomes in a chemically induced rat model of Huntington's disease (HD). This study showed increased expression of key markers associated with medium spiny neurons, including cAMP-regulated phosphoprotein of 32 kDa (DARPP-32), dopamine receptor type 2 (D2R), and brain-derived neurotrophic factor (BDNF) [31]. Biodistribution studies in mice revealed that intravenously administered NestaCell® initially accumulates in the lungs before redistributing to multiple organs, including the central nervous system (CNS). Notably, a small proportion of cells remained detectable in the brain up to 30 days post-infusion, suggesting the potential for sustained therapeutic effects [32]. Toxicology assessment in mice demonstrated good tolerability at doses up to 1 million cells per animal, approximately 40 times higher than those proposed for clinical use. However, further investigations are warranted to access potential thromboembolic risks associated with cellular aggregation [33].

The primary objective of this study was to evaluate the long-term safety and tolerability of intravenously administered NestaCell® in participants with HD. Secondary objectives included a preliminary evaluation of NestaCell® efficacy, based on changes in the Unified Huntington’s Disease Rating Scale (UHDRS) Total Motor Score (TMS) and Total Functional Capacity (TFC). Additionally, the study investigated the immunological effects of NestaCell® administration by monitoring CD4 and CD8 lymphocyte counts, the CD4/CD8 ratio, and levels of the inflammatory cytokines IL-4, IL-6, IL-10, and TNF-α.

## Methods

### Study design

This was a prospective, open-label, single-centre, phase I clinical trial. The Ethics Committee approved the study protocol, and written informed consent was obtained from all participants prior to enrolment.

As recommended by the FDA guidance for early-phase trials of advanced therapy products [34], administration was staggered with intervals of 30 days or more (± 7 days) between participants. The first patient enrolled received hDPSC at a dose of 1 million cells/kg, followed by the second and third patients at monthly intervals. Each received three monthly doses of hDPSC. One month after the third dose in the third patient, the Investigator and the DSMC reviewed the safety data. Following confirmation of safety, enrolment in the 2 million cells/kg cohort began, following the same staggered dosing schedule. Both cohorts were administered three monthly doses of NestaCell® at visits V0, V2, and V4.

The study began with a four-month screening and standardisation period (V-4 to V-1), followed by a 12-month treatment and initial follow-up phase (V0 to V9), and a four-year long-term monitoring phase. After the last enrolled patient completed visit V9, the Investigator, in collaboration with the Data and Safety Monitoring Board (DSMB) and sponsor representatives, conducted a comprehensive risk–benefit assessment. Based on this review, the protocol was amended to include additional dosing during the long-term monitoring period. These consisted of cycles of three consecutive monthly administrations repeated every six months, resulting in six doses per year.

### Study population

Participants met the following inclusion criteria: males aged 21–65 with genetically confirmed Huntington’s disease, characterised by 40–50 CAG repeats on chromosome 4, a UHDRS-TMS of ≥ 5, and a UHDRS-TFC of 8–11.

The exclusion criteria were diagnosis of juvenile Huntington’s disease, epilepsy, major cognitive disorder, active decompensated psychiatric illness, current or past malignancy, severe or uncontrolled medical conditions (other than Huntington’s disease); history of suicide attempt; positive serology for HIV, HTLV, HBV, HCV, or syphilis (FTA-ABS); allergy to contrast agents or bovine-derived products; and current or anticipated use of immunosuppressants.

Participants could withdraw consent and discontinue at any time. Discontinuation might also occur due to loss to follow-up, adverse events including neoplasia, at the Investigator’s discretion, newly identified or emerging exclusion criteria, protocol violations, the Sponsor's decision, death, the use of prohibited concomitant medications, or any clinical, laboratory, or psychological condition deemed incompatible with continued participation. Significant disease progression after additional doses could also warrant discontinuation.

### Intervention

The hDPSCs used in this study were developed through a collaboration between Instituto Butantan, a leading Brazilian research institute, and Cellavita, a Brazilian biotechnology company specialising in regenerative medicine. The investigational product, NestaCell®, consists of stem cells derived from the pulp of deciduous teeth donated by healthy individuals aged 5 to 12 years. The procedure was approved by an ethics committee (approval number 51005115.9.0000.5412), and written informed consent was obtained from both donors and their legal guardians for the use of their teeth in clinical research.

Donor eligibility criteria included full immunisation, no history of blood transfusions, and no high-risk exposure to communicable diseases. Blood samples were collected and tested for transmissible infectious agents, including HCV, HBV, HIV-1/2, HTLV-I/II, Chagas disease, syphilis, CMV, Parvovirus B19, and EBV.

Teeth were processed within 48 h of collection. The cells were isolated from dental pulp and expanded as described elsewhere [35, 36]. At Cellavita, the dental pulp was isolated and transferred to a 12.5 cm^2^ flask (Corning®) for cell culture. The cells were cultivated in DMEM-F12 supplemented with 200 mM L-glutamine (Dulbecco’s Modified Eagle’s Medium, Gibco/Life Technologies), 10–15% fetal bovine serum (HyClone), and 100X MEM NEAA (Gibco/Life Technologies Brasil). In passage zero (0), 50 mg/mL of gentamicin (Sigma Aldrich®) was used. For passages 1 to 5, cultures were maintained with 10,000 U to 10 mg/mL of penicillin/streptomycin (Sigma Aldrich®). The pulp and hDPSCs were incubated at 37 °C in a humidified incubator with 5% CO₂ and 95% air.

Cells were expanded until passage 2, followed by cryopreservation to establish a master cell bank (MCB) at – 150 °C. After thawing, cells were expanded until passage 5, then cryopreserved in liquid nitrogen at – 150 °C to form the active component (AC). The procedure is described in more detail elsewhere [35, 36].

On the day of administration, the AC was thawed, washed, and resuspended in 0.9% saline. The final product was filled in 10- or 20-mL syringes at a concentration of 2 million cells/mL. Each participant received 3–8 syringes depending on body weight and randomisation group. Each syringe was administered slowly, followed by a 10 mL saline flush to ensure complete cell delivery.

Cells from the MCB, the AC, and the final product underwent comprehensive quality control prior to clinical use. Testing of MCB and AC included cell viability, immunophenotyping, quantification of BDNF secretion, in vitro differentiation potential, screening for human viral genes, karyotyping, and testing for sterility, endotoxins, and mycoplasma.

For the final product, due to the limited post-thaw stability of 4 h, quality control included sterility testing, endotoxin and mycoplasma assessment, and visual inspection for cell clumping. Further details on each analysis performed are provided in Table 1.

**Table 1:** The release analyses conducted on master cell banking (MCB), the active component (AC), and NestaCell® product

Production step	Analysis batch release	Analysis details
MCB AC NestaCell®	Viability and cell count	**Method**: Trypan blue 0.4% exclusion **Equipment**: TC20™ Automated Cell Counter
MCB AC	Immunophenotyping	**Method**: Flow cytometry using monoclonal antibodies against CD105, CD90, CD73, CD34, CD45, CD11b, CD19 (BD Biosciences), as well as HLA-DR and Nestin **Equipment**: FACSCalibur (BD Biosciences)
MCB AC	BDNF secretion quantification	**Method**: ELISA using the Human BDNF ELISA Kit (Invitrogen) **Equipment**: SpectroStarNANO (BMG LABTECH)
MCB AC	In vitro differentiation into mesodermal lineages	**Method**: Culture in tissue-culture vessels with lineage-specific differentiation media **Osteogenic Medium**: StemPro® Osteocyte/Differentiation Basal Medium + Osteogenesis Supplement (Gibco) **Chondrogenic Medium**: StemPro® Chondrocyte Differentiation Basal Medium + Chondrogenesis Supplement (Gibco)
MCB AC NestaCell®	Sterility testing	**Method**: Direct inoculation using Fluid Thioglycollate Medium (THIO) and Casein Soy Broth (TSB), both from Merck
MCB AC NestaCell®	Mycoplasma detection	**Method**: Bioluminescence assay using the MycoAlert™ PLUS Kit (Lonza) **Equipment**: GloMax®-Multi Jr Luminometer
MCB AC NestaCell®	Endotoxin testing	**Method**: Limulus Amebocyte Lysate (LAL) assay using Endosafe® nexgen-PTS™ cartridges **Equipment**: Endosafe® nexgen-PTS™
MCB AC	Karyotype analysis	**Method**: Array-CGH using the KaryoNIM® STEM CELLS platform to detect genomic instability
NestaCell®	Cell clumping Assessment	**Method**: Visual inspection of the final product

**Fig. 1:**
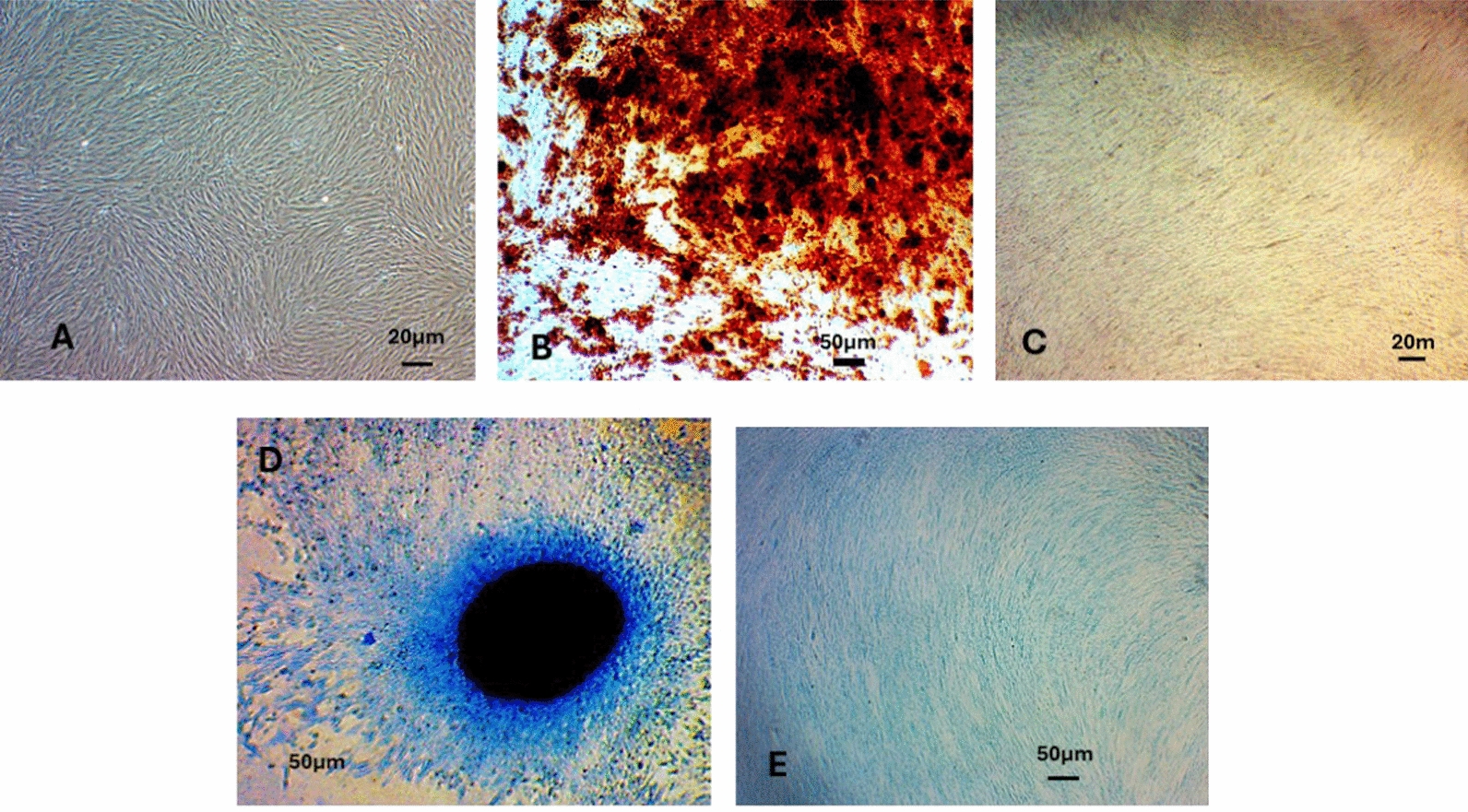
Photomicrographs of human induced dental pulp stem cells (hIDPSCs) at passage 5. **A** hIDPSCs in culture showing fibroblast-like morphology. **B** Osteogenic differentiation demonstrated by Alizarin Red staining following induction with Osteocyte/Differentiation Basal Medium StemPro® (Gibco) supplemented with Osteogenesis Supplement (Gibco). **C** Negative control for osteogenic differentiation. **D** Chondrogenic differentiation evidenced by micromass formation after induction with Chondrocyte Differentiation Basal Medium StemPro® (Gibco) supplemented with Chondrogenesis Supplement (Gibco). **E** Negative control for chondrogenic differentiation. Magnification: 40 × for panels A and B; 10 × for panels **C, D**, and **E**

Phenotypic characterisation confirms the expression of CD105, CD90, and CD73 (≥ 90%) and Nestin (≥ 70%), while lacking expression of CD34, CD45, CD11b, and CD19 (≤ 2%), consistent with the profile of mesenchymal stem cells [37]. Low expression of HLA-DR (≤ 2%) minimises the risk of immunogenicity*.* These cells exhibit a fibroblast-like morphology in vitro, with a viability rate of 70% or higher [38]. The hDPSCs demonstrate in vitro potential for chondrogenic and osteogenic differentiation (Fig. 1) and secrete Brain-Derived Neurotrophic Factor (BDNF) at concentrations ≥ 1 ng/mL.

The NestaCell® manufacturing process complies with Good Manufacturing Practices (GMP) as required by the Brazilian Regulatory Authority and is protected by a US patent, US 20160184366 A. Genetic stability is confirmed throughout production via karyotyping using comparative genomic hybridization (CGH). The cells are sterile, with no detectable bacteria, fungi, or mycoplasma, and exhibit endotoxin levels below 5 EU/kg.

### Safety assessment

Vital signs were monitored for 48 h after administration, and physical examinations were recorded at each visit. Blood and urine analyses were performed at all visits, with additional collections at 24- and 48-h post-administration during visits V0, V2, and V4. Immunogenicity samples (CD4 + and CD8 + T cells) were collected before administration and at 12-, 24-, and 48-h post-administration. Screening for neoplasia occurred at visits V-4 and V-8 and annually at visits V-10, V-11, V-12, and V-13.

## Results

### Study population

**Fig. 2:**
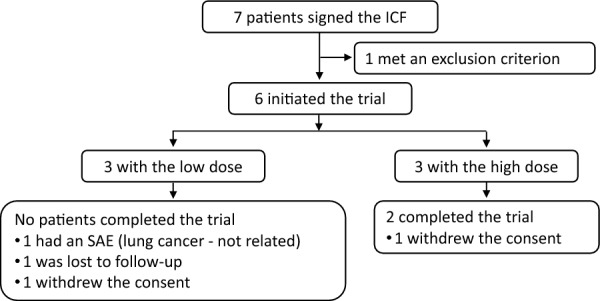
Study flowchart. ICF: informed consent form

Seven patients were screened for this trial, and six were subsequently enrolled. All enrolled participants completed a two-year follow-up; five completed three-year, four completed four-year, and two participants completed the entire five-year trial. Four patients discontinued prematurely: one due to a serious adverse event (SAE) at the 1 million cells/kg dose (lung cancer, discontinued by the investigator), one was lost to follow-up, and two patients withdrew his consent (Fig. 2). The patient’s baseline demographics and UHDRS scores are summarised in Table 2.

**Table 2:** Demographic baseline characteristics (V-1) per patient

Characteristics	Patients						
	1	2	3	4	5		6
	1 million/kg			2 million/kg			
Age (years)	42	51	50	58	40		49
BMI (kg/m^2^)	22.6	27.1	25.1	27.1	28.5		26.1
Education (years)	0–8	0–8	13–21	13–21	9–12		13–21
CAG repeats	46	44	43	42	46		42
CAP score	107.2	113.9	103.7	111.0	102.1		93.8
Years of symptoms	7	5	5	5	3		6
UHDRS-TMS	59	46	13	17	11		44
UHDRS-TFC	7	11	11	13	11		6

BMI: Body mass index. CAG: Cytosine-Adenine-Guanine. CAP score: an indicator combining CAG and age. The UHDRS-TMS and UHDRS-TFC are dimensionless scales

### Exposure

Table 3 summarises patient’s exposure to NestaCell®, comprising 102 administrations: 31 at the 1 million cells/kg dose and 71 at the 2 million cells/kg dose. P3 received a single long-term administration period, and after 21 days, the treatment was discontinued.

**Table 3:** Exposure to the investigational product, as determined by the number of NestaCell® administrations

Patient	Original	Amendment	Total
P1	3 × 1 million/kg	12 × 1 million/kg	15 × 1 million/kg
P2	3 × 1 million/kg	9 × 1 million/kg	12 × 1 million/kg
P3	3 × 1 million/kg	1 × 1 million/kg*	4 × 1 million/kg
P4	3 × 2 million/kg	23 × 2 million/kg	26 × 2 million/kg
P5	3 × 2 million/kg	16 × 2 million/kg	19 × 2 million/kg
P6	3 × 2 million/kg	23 × 2 million/kg	26 × 2 million/kg

### Safety

Treatment-emergent adverse events (TEAEs), categorised by intensity and relationship to the investigational product, are shown in Table 4, while all reported TEAEs are detailed in Table 5.

**Table 4:** Treatment-emergent adverse events (TEAEs) by causality, intensity, and severity

	1 million/kg		2 million/kg		Treated	
	N	n (%)	N	n (%)	N	n (%)
Total TEAEs	21	3 (100.0)	26	3 (100.0)	47	6 (100.0)
Treatment-related TEAEs	2	1 (33.3)	4	2 (66.7)	6	3 (50.0)
Serious TEAEs	1	1 (33.3)	0	0	1	1 (16.7)
Related severe TEAEs	0	0	0	0	0	0
TEAEs intensity	21	3 (100.0)	26	3 (100.0)	47	6 (100.0)
Mild	11	3 (100.0)	12	3 (100.0)	23	6 (100.0)
Moderate	8	3 (100.0)	14	3 (100.0)	22	6 (100.0)
Severe	2	1 (33.3)	0	0	2	1 (16.7)
TEAEs that resulted in treatment withdrawal	1	1 (33.3)	0	0	1	1 (16.7)
TEAEs that resulted in death	0	0	0	0	0	0

TEAEs—Treatment-emergent adverse events. N = number of TEAEs. n = number of patients. () = percentage of patients within the group

**Table 5:** Treatment-emergent adverse events (TEAEs) by affected organ class, as defined by the Medical Dictionary for Regulatory Activities (MedDRA, Version 24.1, September 2021)

TEAEs by system organ class	1 million/kg		2 million/kg		Treated	
	N	n (%)	N	n (%)	N	n (%)
Number of TEAEs	21	3 (100.0)	26	3 (100.0)	47	6 (100.0)
Congenital, familial and genetic	0	0	2	2 (66.7)	2	2 (33.3)
Endocrine	0	0	1	1 (33.3)	1	1 (16.7)
Gastrointestinal	2	2 (66.7)	1	1 (33.3)	3	3 (50.0)
Infections and infestations	4	3 (100.0)	1	1 (33.3)	5	4 (66.7)
Injury, poisoning and procedural complications	0	0	2	1 (33.3)	2	1 (16.7)
Investigations	3	3 (100.0)	2	2 (66.7)	5	5 (83.3)
Musculoskeletal and connective tissue	1	1 (33.3)	4	2 (66.7)	5	3 (50.0)
Neoplasms benign, malignant and unspecified	1	1 (33.3)	0	0	1	1 (16.7)
Psychiatric	4	3 (100.0)	5	3 (100.0)	9	6 (100.0)
Renal and urinary	1	1 (33.3)	0	0	1	1 (16.7)
Reproductive system and breast	2	2 (66.7)	0	0	2	2 (33.3)
Respiratory, thoracic and mediastinal	1	1 (33.3)	2	2 (66.7)	3	3 (50.0)
Skin and subcutaneous tissue	2	1 (33.3)	5	2 (66.7)	7	3 (50.0)
Surgical and medical procedures	0	0	1	1 (33.3)	1	1 (16.7)

TEAEs—Treatment-emergent adverse events. N = number of TEAEs. n = the number of patients. () = percentage of patients within the group

During the first year of the study, patients were closely monitored in an Intensive Care Unit (ICU) for 48 h following each administration. No adverse events (AEs) were observed during the ICU monitoring periods following 18 administrations, with six patients receiving three injections each.

Over the five-year follow-up, 47 treatment-emergent adverse events (TEAEs) were reported after hospital discharge. Of these, six were deemed related to treatment by the investigator: two at the 1 million cells/kg dose and four at the 2 million cells/kg dose. All involved temporary changes in hair pigmentation or regrowth. These symptoms were noticed approximately one month after the infusion and persisted for an additional month.

Forty-one TEAEs, including the serious adverse event (SAE) described below, were considered by the investigator to be unrelated to NestaCell. These TAEs were evenly distributed across dose groups and primarily attributed to the progression of Huntington’s disease (HD). The most frequently reported system organ class was psychiatric disorders (nine events), including alcoholism, anxiety, depression and depressive symptoms, affective disorder, and insomnia.

The second most frequently affected organ/system was skin and subcutaneous tissue, with seven reports: one case of contact dermatitis (unrelated) and the six hair-related events.

One SAE occurred in P3, a 53-year-old male who received the 1 million cells/kg dose. This patient, a heavy smoker, had an asymptomatic pre-existing pulmonary nodule identified on baseline CT imaging. Following a pulmonology review, the lesion was considered likely benign, and after a benefit-risk assessment, the patient was authorised for enrolment.

Twenty-nine months after enrolment—following four NestaCell® administrations (three as per protocol and one under an amendment)—routine follow-up imaging revealed growth of the pulmonary nodule. A bronchoscopic biopsy confirmed primary pulmonary adenocarcinoma, leading to study discontinuation and tumour surgical removal. Genetic analysis of the excised tissue revealed no markers associated with NestaCell®, indicating that there was no graft-derived material in the tumour. This previously published case [39] represents the first documented instance of stem cell administration in a patient with a pre-existing tumour. At the time of this report submission, the patient remains cancer-free, and the event was assessed as unrelated to NestaCell® administration.

A TEAE of severe intensity also occurred in P3, involving worsening depressive symptoms over 93 days. The event’s relationship to the investigational product was considered indeterminate. Psychiatric manifestations, including depression, are common in HD and may reflect the natural course of the disease. However, the contribution of the investigational product could not be excluded.

Patients P2, P3, and P5 experienced mild, clinically asymptomatic elevations in alanine aminotransferase (ALT) and aspartate aminotransferase (AST), considered unrelated to cell therapy and likely due to concurrent polypharmacy. Patients P4 and P6 exhibited minor fluctuations in CD4 and CD8 lymphocyte counts throughout the study period. P4 showed CD4 counts ranging from 631–1593 cells/mm^3^ (mean: 1,107 ± 234 cells/mm^3^) and CD8 counts from 188 to 567 cells/mm^3^ (mean: 337 ± 89 cells/mm^3^). P6 demonstrated CD4 counts ranging from 541 to 1184 cells/mm^3^ (mean: 835 ± 156 cells/mm^3^) and CD8 counts from 264 to 651 cells/mm^3^ (mean: 442 ± 129 cells/mm^3^). Notably, both patients maintained CD4/CD8 ratios within normal reference ranges (0.98–3.24) throughout the study, with P4 showing consistently elevated ratios (mean: 3.28 ± 0.37) and P6 maintaining ratios closer to the lower normal range (mean: 1.88 ± 0.22). All fluctuations remained within normal laboratory reference values and showed no consistent pattern of immunosuppression or activation.

Cytokine monitoring revealed persistently elevated IL-4 in P6, potentially attributable to HD progression or allergic response, and a transient elevation of IL-6 in P5, not associated with infection and likely reflective of disease progression.

Vital signs and electrocardiographic parameters generally remained within normal limits, with isolated minor deviations assessed as non-clinically significant and unrelated to cell therapy.

### Efficacy

Efficacy outcomes were analysed over two periods: the first year of study visits (V0 to V9) and the long-term period, from the first administration of the extended dosing phase until the trial's final visit. Annualised slopes were calculated individually for each participant and outcome measure.

**Fig. 3:**
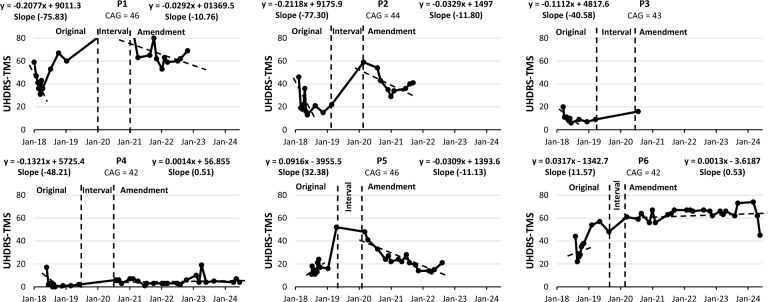
Individual UHDRS-TMS throughout the study. P1, P2, and P3 received 1 million cells/kg; P4, P5, and P6 received 2 million cells/kg. Lower TMS values indicate better motor outcomes. Original: first year of the study, from V0 to V9. Interval: period between V9 and the first NestaCell® administration under the protocol amendment. Amendment: from the first administration in the long-term extension period to the final visit

UHDRS-TMS slopes varied distinctly across participants. During the initial period, patients P1, P2, P3, and P4 exhibited negative slopes, indicating clinical improvement, while P5 and P6 showed positive slopes, suggesting clinical decline. In the long-term period, P1 and P2 maintained favourable negative slopes. P4 shifted to a slightly positive slope, whereas P5 transitioned to a negative slope compared to their initial positive slope. P6 continued with a positive slope in the long term, although less steep than in the initial phase. P3 received only one additional dose before discontinuation, resulting in insufficient data points for slope calculation (Fig. 3).

**Fig. 4:**
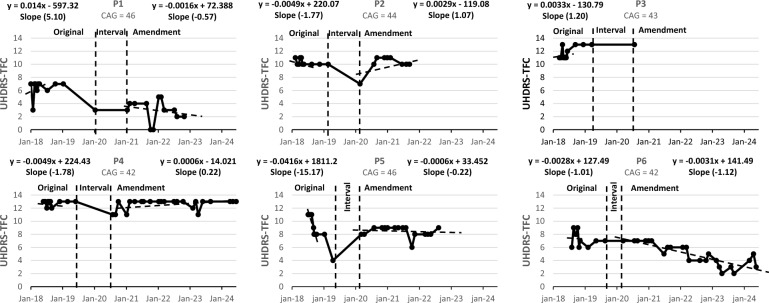
Individual UHDRS-TFC throughout the study. P1, P2, and P3 received 1 million cells/kg; P4, P5, and P6 received 2 million cells/kg. Higher TFC values indicate better functional outcomes. Original: first year of the study, from V0 to V9. Interval: period between V9 and the first NestaCell® administration under the protocol amendment. Amendment: from the first administration in the long-term extension period to the final visit

UHDRS-TFC slopes were positive in the initial period for P1 and P3, reflecting a favourable clinical response, and negative for the remaining patients. In the long-term period, positive TFC slopes were observed for P2 and P4, while negative slopes persisted for the other three evaluated patients. PatientP3's early discontinuation prevented slope calculation during the long-term phase (Fig. 4).

## Discussion

This first-in-human clinical trial aimed to evaluate the safety and tolerability of NestaCell® in six patients with Huntington's disease. While the protocol was initially planned for three administrations, it was amended during the second year to allow up to 26 administrations over a five-year follow-up. This change was prompted by encouraging short- and medium-term safety outcomes, as well as preliminary evidence of efficacy. To our knowledge, this represents one of the highest reported exposures to stem cell therapy.

No short-term adverse events (AEs) were observed during the 48 h of intensive multiparametric monitoring, nor were any reported within the first 15 days. Among the 47 treatment-emergent adverse events (TEAEs) documented during medium-term (16–180 days) and long-term (> 180 days) follow-up, 41 were considered unrelated to NestaCell and were attributed instead to incidental causes or disease progression. Six TEAEs, characterised by transient changes in hair pigmentation (darkening) or hair regrowth, were assessed as treatment related.

One serious adverse event (SAE), deemed unrelated to NestaCell®, occurred in a patient from the 1 million cells/kg dose group. This involved the growth of a pre-existing pulmonary nodule identified during screening, leading to the patient's early discontinuation at the two-year visit (V10). Genetic analysis confirmed the absence of NestaCell engraftment, further supporting the investigational product’s non-tumorigenic profile [39].

Some events observed in this study, such as mild elevations in ALT and AST in Patients 2, 3, and 5, were likely due to polypharmacy and not related to stem cell therapy, as they were asymptomatic and resolved without clinical significance. To our knowledge, there is currently no direct evidence linking MSC therapy to adverse effects specifically attributed to polypharmacy.

MSCs are known to modulate immune responses by reducing CD4⁺ and CD8⁺ T cell activation and promoting regulatory phenotypes. The hDPSCs, as a subset of MSCs, similarly suppress T cell proliferation, influence macrophage polarisation, and reduce inflammation via cytokine secretion [40–43]. However, these immunomodulatory effects are context-dependent and influenced by factors such as the inflammatory environment, dosing, and patient variability [44, 45].

In our study, minor fluctuations in CD4/CD8 lymphocyte counts in patients P4 and P6 contrasted with the typical immunosuppressive effects of hDPSCs. Notably, the short-term response within 24 h post-infusion was variable, with some participants showing increases and others decreases (see supplementary data). Several factors may account for this.

First, MSC immunomodulation is dose-dependent and may follow a biphasic pattern, where lower doses are immunostimulatory and higher doses immunosuppressive [46, 47]. Both patients received the higher dose (2 million cells/kg), possibly contributing to a different response. Second, MSCs display functional plasticity, shifting between pro-inflammatory (MSC1) and anti-inflammatory (MSC2) phenotypes in response to local cytokines [48, 49]. In chronic neuroinflammation, as seen in Huntington’s disease, the microenvironment may shape MSC behaviour and T cell responses [50].

These fluctuations may also reflect normal biological variability, as both patients-maintained lymphocyte counts within reference ranges and stable CD4/CD8 ratios over one year [51]. No clinical signs of immunosuppression or autoimmunity were observed. Similar immune variability without adverse effects has been reported in other long-term MSC studies [52, 53]. ADDIN EN.CITE [54]

Overall, these findings highlight the notable tolerability of NestaCell®, even with extensive exposure. Both administered doses demonstrated potential clinical benefits as reflected by stability or improvement in UHDRS-TMS and UHDRS-TFC.

## Conclusion

NestaCell® demonstrated a favourable safety profile, with no serious treatment-related adverse events, even after extensive exposure over a five-year period. Minor events, such as temporary changes in hair pigmentation, were well-tolerated. No signs of tumorigenicity or significant immunogenic responses were observed. Clinical benefits, including stability or improvement in UHDRS-TMS and UHDRS-TFC, were noted, supporting further investigation in more extensive trials to confirm efficacy and long-term benefits.

## Data Availability

The datasets generated and analysed during the present study are available from the corresponding author upon reasonable request.

## Abbreviations

BDNF: Brain-derived neurotrophic factor
BMI: Body mass index
CAG: Cytosine-adenine-guanine
CNS: Central nervous system
D2R: Dopamine receptor type 2
DARPP-32: Dopamine- and cAMP-regulated neuronal phosphoprotein
DTI: Diffusion tensor imaging
ECG: Electrocardiogram
GMP: Good manufacturing practices
HD: Huntington disease
HTT: Huntingtin gene
hDPSDs: Human dental pulp stem cells
ICF: Informed consent form
ICU: Intensive care unit
ITT: Intention-to treat
MHC: Major histocompatibility complex
MSC: Mesenchymal stem cell
PCR: Polymerase chain reaction
SAE: Serious adverse event
TEAE: Treatment-emergent adverse events
TFC: Total functional capacity
TMS: Total motor score
UHDRS: Unified Huntington’s disease rating scale

## Declarations

### Clarification

The investigational product evaluated in this study was originally developed under the name Cellavita HD. In March 2020, it was registered under the trademark NestaCell®. Both names refer to the same cell-based product. In this manuscript, the current designation NestaCell® is used consistently; however, the original name Cellavita HD is retained when citing regulatory documents—such as the ethics committee approval—where that designation was originally employed.
